# Modeling the synergistic antibacterial effects of honey characteristics of different botanical origins from the Sahara Desert of Algeria

**DOI:** 10.3389/fmicb.2015.01239

**Published:** 2015-11-06

**Authors:** Hadda Laallam, Larbi Boughediri, Samia Bissati, Taha Menasria, Mohamed S. Mouzaoui, Soumia Hadjadj, Rokia Hammoudi, Haroun Chenchouni

**Affiliations:** ^1^Laboratoire de Bioressources Sahariennes, Université Kasdi Merbah OuarglaOuargla, Algeria; ^2^Equipe de Palynologie, Laboratoire de Biologie Végétale, University of AnnabaAnnaba, Algeria; ^3^Department of Applied Biology, Faculty of Exact Sciences and Natural and Life Sciences, University of TebessaTebessa, Algeria; ^4^Laboratoire Régional du Centre Algérien du Contrôle de la Qualité et de l'EmballageOuargla, Algeria; ^5^Laboratoire de Protection des Ecosystèmes en Zones Arides “EcoSys,” Université Kasdi Merbah OuarglaOuargla, Algeria; ^6^Laboratory of Biogeochemistry of Desert Environments, Faculty of Natural and Life Sciences, University of Kasdi Merbah OuarglaOuargla, Algeria; ^7^Department of Natural and Life Sciences, Faculty of Exact Sciences and Natural and Life Sciences, University of TebessaTebessa, Algeria; ^8^Department of Ecology and Plant Biotechnology, Faculty of Natural and Life Sciences, University of Batna 2Batna, Algeria

**Keywords:** honey characterization, antibacterial effects, floral origin, Sahara Desert bioresources, GLMM, antibacterial chemotherapy

## Abstract

**Background:** Honey has multiple therapeutic properties due to its composition with diverse components.

**Objectives:** This study aims to investigate the antimicrobial efficacy of Saharan honeys against bacterial pathogens, the variation of honey floral origins, and its physicochemical characteristics.

**Materials and Methods:** The antimicrobial activity of 32 samples of honey collected from the Algerian Sahara Desert was tested on four bacteria; *Bacillus subtilis, Clostridium perfringens, Escherichia coli*, and *Staphylococcus aureus*. The botanical origin of honeys and their physicochemical properties were determined and their combined antibacterial effects were modeled using a generalized linear mixed model (GLMM).

**Results:** Out of the 32 study samples, 14 were monofloral and 18 were multifloral. The pollen density was on average 7.86 × 10^6^ grains/10 g of honey, water content was 14.6%, electrical conductivity (EC) was 0.5 μS/cm, pH was 4.38 ± 0 50, hydroxymethylfurfural (HMF) content was 82 mg/kg of honey, total sugars = 83%, reducing sugars = 71%, and the concentration of proline = 525.5 ± 550.2 mg/kg of honey. GLMM revealed that the antibacterial effect of honey varied significantly between bacteria and floral origins. This effect increased with increasing of water content and reducing sugars in honey, but it significantly decreased with increase of honey EC. *E. coli* was the most sensitive species with an inhibition zone of 10.1 ± 4.7 mm, while *C. perfringens* was the less sensitive. Honeys dominated by pollen of Fabaceae sp. were most effective with an overall antimicrobial activity equals to 13.5 ± 4.7 mm.

**Conclusion:** Saharan honeys, of certain botanical origins, have physicochemical and pollinic characteristics with relevant potential for antibacterial purposes. This encourages a more comprehensive characterization of honeys with *in vivo* and *in vitro* investigations.

## Introduction

In recent years, pathogenic microorganisms have developed multiple drug resistance due to the abundant and wide spared use of antimicrobial drugs that were commonly used in human medicine (Al-Waili et al., 2011; Noori et al., 2013). Even with the broad spectrum of some antibacterial agents, the choice of most suitable remains relatively limited due to the development of bacterial resistance, breakthrough infections, and ever-increasing therapeutic problem (Shahid et al., 2008). Alternative antimicrobial strategies are therefore urgently needed using various natural, traditional, and nonconventional sources (Al-Waili et al., 2011; Lucera et al., 2012). Antimicrobial substances originated from natural resources have been widely exploited for this purpose, with a specific focus of studies on a specific product “Honey” due to a long tradition of use within various medical and food systems (Lusby et al., 2005; Al-Waili et al., 2011). Honey is used to treat certain topical infections and even for accelerating wound healing and epithelization (Simon et al., 2006; Mandal and Mandal, 2011).

Honey is used for centuries and still widely used as an antiseptic where its main characterized role is the prevention and limitation of bacterial infection derived largely from biochemical properties related to peroxide generation via glucose oxidase activity (Brudzynski, 2006), nonperoxide effect such as, osmolarity, acidity, aromatic acids, phenolic, and other phytochemical compounds such as methylglyoxal (Mundo et al., 2004; Lusby et al., 2005; Lee et al., 2008; Mavric et al., 2008). Moreover, honey serves as a natural antioxidant and a rich source of minerals, carbohydrates, proteins, and vitamins with nutraceutical and probiotic properties (Bertoncelj et al., 2007; Begum et al., 2015).

In addition, the antibiotic and antiseptic effects of honey have been scientifically proven in several studies (Shamala et al., 2002; Werner and Laccourreye, 2011). These effects are mainly due to the bio-chemical composition of honey that contains high sugar and low water concentrations with low pH. These properties generate the high osmolarity that produces the antimicrobial action (Wahdan, 1998). Honey also contains molecules inhibiting bacterial growth, such as hydrogen peroxide produced by glucose oxidase; and also the non-peroxide inhibins also known as phytochemicals composed (Cushnie and Lamb, 2005; Adeleke et al., 2006; Bell, 2007; Montenegro and Mejías, 2013).

It is noteworthy to mention that different analysis techniques of honey components may be implemented. The analysis of some of these substances requires special and sophisticated methods such as those performed using spectrophotometric assays, particularly gas chromatography-mass spectrometer (GC-MS), liquid chromatography-mass spectrometer (LC-MS), and nuclear magnetic resonance (NMR). These techniques are used to assess contents of molecules and elucidate the structure of active molecules (Bertoncelj et al., 2007; Tiwari et al., 2014, 2015).

The antimicrobial activities of honey have been extensively investigated against a large category of bacterial and fungal pathogens including *Staphylococcus aureus, S*. *pyogenes, S*. *mutans, Bacillus cereus, Listeria monocytogens, Escherichia coli, Klesiella pneumonia, Pseudomonas aeruginosa*, and *Candida albicans* (Mundo et al., 2004; Basualdo et al., 2007; Lee et al., 2008; Sherlock et al., 2010; Estevinho et al., 2011). The differences reported in antimicrobial effects of honey are dependent on its geographical origin thus the botanical source as well as time and processing harvesting, storage conditions, and the nature of pathogens tested (Sherlock et al., 2010; Al-Waili et al., 2011).

Since the antibacterial activity of honey varies depending on the floral origin (Salomon, 2010; Alzahrani et al., 2012), many studies investigated the biochemical composition and pollen contents of honeys of different melliferous plant species to determine levels of natural antibiotic compounds (e.g., Lins et al., 2003; Muñoz et al., 2007). The content of these inhibins in honey pollens depends on the plant species from which they originate in other words according to the floral origin. However, few studies examined the physicochemical composition and antibiotic properties of honey whose floral origin is derived from plant species living under extreme environmental conditions such as the Sahara Desert.

Although the honeybee (*Apis mellifera*) is known as a polylectic species (Reybroeck et al., 2014), honey harvested from the Saharan regions show great variability in pollen composition and density (Noori et al., 2013). This is mainly due to local ecological and floristic characteristics where the hive was installed. Moreover, under the environmental conditions of desert, diversity and abundance of plants are low (Bradai et al., 2015); so that the bee produces two honey categories (i) monofloral honey (i.e., mainly dominated by the pollen of one plant) in regions very little diversified in melliferous plants, or (ii) a multifloral honey (i.e., containing mixed pollen origin) when melliferous plants are diversified and abundant (Von der Ohe et al., 2004). This difference in the pollen composition in honey may result from the flowering period of plants (Campos et al., 2008). Additionally to this, the elective factor that the bee can exercise among the available melliferous plants should be considered (Reybroeck et al., 2014).

Given these facts, the aim of this study is based on the following question: do the botanical origin and pollen composition of honey affect its antibiotic properties and therefore its antibacterial potency? Moreover, the study seeks to determine if differences in physicochemical composition of honey can induce a different impact on its antimicrobial activity against pathogenic bacteria, with taking into account the botanical origin differences. Hence the interest of a physicochemical and pollen analysis of honeys of the Sahara which proves not only important to characterize these honeys and determine their floral origin, but also to determine the most effective floral origin and the parameters that have more antimicrobial effect against the bacteria tested.

## Materials and methods

### Choice and collection of honey samples

**Figure 1:**
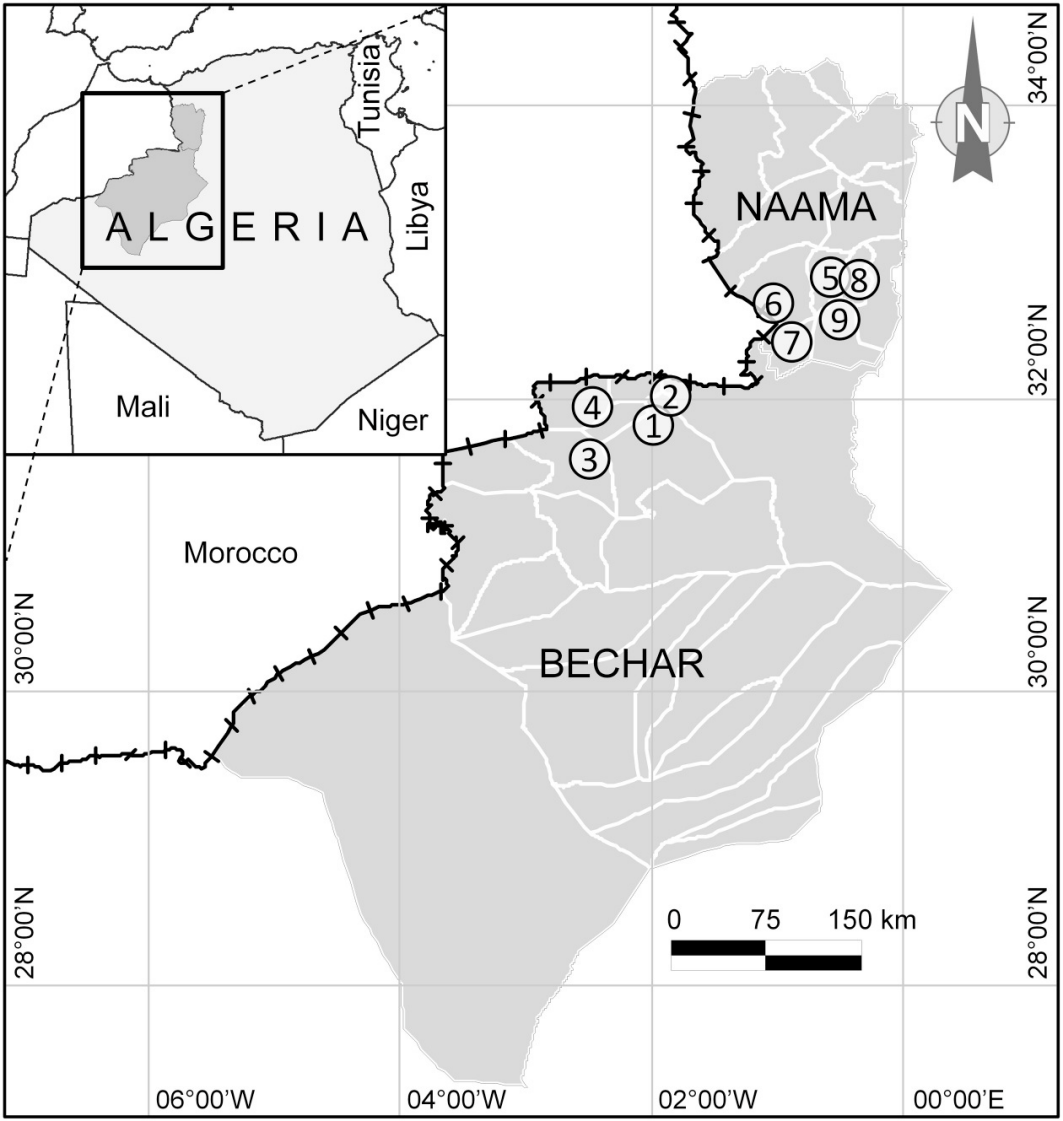
**Geographic location of the study area including honey harvesting sites (solid circles) in the region of Bechar (1, Benzireg; 2, Beni Ounif; 3, Djedid; 4, Sfissifa) and Naama (5, Ain Safra; 6, El Hamar; 7, Djneine; 8, Tiout; 9, Moghrar) located in the Desert Sahara of Algeria**.

Honey samples of the current study were collected from nine localities in southwestern Algeria, in the region of Naama and Bechar located in the Algerian Sahara (Figure 1), where the prevailing climate is hot arid. People of the Sahara, as well as the Algerian populations in general, frequently use honey as a cure for several diseases due to its multiple healing properties (Boukraâ, 2013), specifically the higher efficacy of the Saharan honey compared to that of North Africa (Boukraa and Niar, 2007). Undoubtedly, the spread of the use of honey in traditional and modern medicine has origins linked to the religious beliefs of Muslim people, where many Koranic and Islamic texts reveal that honey is a proven remedy.

A total of 32 honey samples, 20 from Bechar and 12 from Naama, were recovered from local beekeepers just after honey extraction. Each sample was preserved under low temperature before processing to various analyzes in the laboratory.

### Pollinic analysis

The pollen analysis of honeys consisted of two steps following the method of Crompton and Wojtas (1993). The first is the identification of pollen grains observed, whereas the second step was devoted to their count. All honey samples were analyzed without coloring. This allows showing the pollen grain in its natural color with its true appearance for facilitate the identification. Observations were carried out under an optical microscope at a magnification × 100.

Pollen was identified based on comparisons of the observed grains with those known in references. The latters are microscopic preparations of reference that we set up ourselves from fresh anther of local plants and with the help an Atlas of Microphotography (Reille, 1995).

For each sample of honey, the number of pollen grains in 10 g of honey was first counted and the results of that count were then classified in ascending order from I to V (see details in Yang et al., 2014). In parallel, this counts allowed us to make a classification of botanical taxa identified into frequencies; which determines if the honey in question comes from either (i) multiple plants pollinated by bees, so without a clear predominance of a particular plant (multifloral honey), or (ii) otherwise, honey is classified as monofloral (syn. unifloral) in which pollen grains of one plant species dominate (Von der Ohe et al., 2004).

### Physicochemical analysis of honeys

For curative purposes and to benefit from this natural remedy, it is recommended to use fresh and natural honey (Bogdanov and Blumer, 2001). Since antiseptic and antibiotic substances tend to disappear—or at least to be less active—in old honeys (Lobreau-Callen et al., 2000), it is therefore required to proceed prior to physicochemical analyzes of quality control to be able to bind honey features with its microbiological activity. This concerns particularly water and sugar contents, pH, hydroxymethylfurfural (HMF), and obviously proline (Helrich, 1990; Bogdanov et al., 2002). Thus, each honey sample had undergone some physicochemical analyzes:

- **Water content (WC):** expressed in %, is determined using a refractometer for measuring the refractive index at 20°C with reference to the table Chataway according to the method followed by Bogdanov et al. (2002).
- **pH:** was determined by a pH meter on a solution composed of 10 g of honey and 75 mL of distilled water (Bogdanov et al., 2002).
- **Electrical conductivity (EC):** measured (in μS/cm) using a conductimeter device at 20°C of the test solution that consisted of 20% honey weighed as dry matter dissolved in distilled water and brought to a volume of 1/5 (Bogdanov et al., 2002).
- **Hydroxymethylfurfural (HMF):** was measured using Winkler's method (Bogdanov et al., 1999). HMF content is expressed in mg per 1 kg of honey. The HMF is a product of the degradation of fructose and glucose by intramolecular dehydration (Nombré et al., 2010). This parameter is used to control the freshness and quality of honey; thereby a value greater than 60 mg/kg indicates an old honey or the latter has undergone heat treatment degrading its properties (Oddo et al., 1999). Determining the HMF content is based on the measurement of absorbance by spectrophotometry at a wavelength of 550 nm in the presence of barbituric acid and para-toluidine.
- **Total sugars:** sugars represent the largest part of the dry matter of the bee's honey (*Apis mellifera*). Their analysis comes forth by refractometer, which is a quick and simple method (Helrich, 1990).
- **Reducing sugars:** The amount of total reducing sugars, expressed in% from total sugars, was determined titrimetrically according to the volumetric method (Helrich, 1990).
- **Proline:** The proline content (mg/kg honey) was determined using the colorimetric assay with ninhydrin following the method of Ough (1969) defined by Bogdanov et al. (2002). The proline content provides useful information on the maturity of honey and therefore can be used to detect forgeries. It is considered that honey is mature when its proline content is greater than 183 mg/kg. Lower values indicate a lack of maturity or honey falsification (Meda et al., 2005).

### Antibacterial disc diffusion assays

The antibacterial activities of honeys were tested using the agar disc diffusion against four pathogens and resistant bacterial strains, namely: *E. coli* (ATCC25922), *S. aureus* (ATCC25923), *Clostridium perfringens*, and *Bacillus subtilis*. Pure strains of *C*. *perfringens* were provided by the microbiological laboratory of the hospital Mustapha Chaabani (Golea, Ghardaia, Algeria). While *B*. *subtilis* has been isolated from human feces and identified at the Microbiology Laboratory of Bachir Ben Nacer Hospital in El Oued (Algeria), using conventional phenotypic identification protocols.

*C. perfringens* was cultivated using anaerobic jars (GasPak system), whereas other bacteria were grown and purified on nutrient agar (NA). Bacterial inoculum suspensions containing 10^6^–10^8^ CFU/mL were prepared in sterile saline (0.9 g/L) and spread on Mueller-Hinton (MH) agar plates for each strain. Using sterile forceps, Whatman's filter discs (Ø = 5 mm), impregnated with different honeys were placed on the inoculated plates and left at 4°C for 2 h to allow the diffusion before being incubated at 37°C for 24 h. The clear inhibition zones around the discs indicated the presence of antibacterial activity of honey (Harley et al., 2010) which was measured as zone diameter in mm excluding the diameter of disc. Experiments were carried out in triplicates.

To control the susceptibility profile of the Gram-negative bacterium *E. coli* and the Gram-positive bacterium *S. aureus*, standard antibiotic discs were tested using the agar diffusion technique (EUCAST, 2013). The following antimicrobials amoxicillin/clavulanic acid (20/10 μg), gentamicin (15 μg) and trimethoprim-sulfamethoxazole (co-trimoxazole) (1.25/23.75 μg) were applied for both bacteria, whereas amikacin (30 μg), colistin (50 μg), and imipenem (10 μg) were tested against *E. coli*, and ampicillin (10 μg), cefotaxime (30 μg), clindamycin (2 IU), erythromycin (15 IU), fusidic acid (10 μg), spiramycin (100 μg), streptomycin (10 IU) against *S. aureus*.

Standard antibiotic discs of trimethoprim-sulfamethoxazole “SXT” (1.25/23.75 μg per disc) served as a positive control. This combination antimicrobial agent was tested on the Gram-negative bacterium *E. coli* and the Gram-positive bacterium *S. aureus*. The sterile H_2_O served as a negative control in order to determine the minimum inhibitory concentration (MIC) of the study honeys. For that, each honey sample was used to prepare solutions of different proportion (w/v): 5, 10, 15, 20, 25, and 75%. However, no antibacterial activity was observed for all these honey dilutions. Consequently, we only analyzed data related to honey at natural state i.e., used without dilution. Despite that, we focused on the objective of characterizing Saharan honeys in relation with their diverse floral origins.

### Modeling the synergistic antibacterial effects of honey features

As most of studies attributed the antibacterial effects of honey particularly to high sugar content, low water content, low pH and high concentration of flavonoids (Wahdan, 1998). However, these parameters furthermore vary following the botanical origins of honey and the ecological factors that influence both melliferous plants likewise the behavior, physiology and fitness of bees (Reybroeck et al., 2014). Therefore several (biotic and abiotic) parameters are involved in the variation of the honey quality and thus its antimicrobial activities, which makes taking into account all these variables to modeling its antibiotic and antiseptic activities a real challenge to achieve, specifically when it is tested against several pathogens that may react differently.

Accordingly, we used as much as relevant variables of the study honeys in a single statistical model to explain how the antimicrobial activity (diameter of inhibition zone) varies following: floral origins (pollens parameters), physicochemical characteristics of honey, and tested bacteria. We included all the study bacteria in a single model as honey is usually applied to heal diseases caused by the mixture of pathogenic bacteria. Linear mixed-effects models represent the best fit to this kind of data (Pinheiro et al., 2015). In addition, we added to the model other parameters that ensures that the honey is natural with high quality such HMF and proline which provides information about the maturity of honey (Bogdanov et al., 2002). Finally, because samples of honey were collected from different sites (several samples from the same site) in the Sahara Desert, we used generalized linear mixed model (GLMM) to deal with pseudo-replications.

### Statistical analyses and modeling procedures

The values of the physicochemical parameters of the studied honeys were summarized for each botanical origin as means ± standard deviations (SD) and the range (min and max) of observations. The variation of each parameter between botanical origins was tested by the analysis of variance (One-way ANOVA) using the software R (R Core Team, 2015). Multiple comparisons of means (Tukey HSD tests) were performed afterward each ANOVA to distinguish homogeneous groups among botanical origins.

Descriptive statistics (mean, SD, and quartiles) of the antimicrobial activity were computed for each floral origin and bacterial strain based on replicates of honey samples containing that floral origin. Computations were performed using the function “numSummary” in R (R Core Team, 2015) and plotted using the package “ggplot2” (Chang, 2013).

The variation in antibacterial activity was modeled using a mixed-effects modeling procedure in R. The library “nlme” was used to test the effects of bacterial strains, floral origins as well as physicochemical parameters of honey on the dependent variable “inhibition zone.” The categorical factors (bacterial strains and floral origins) and all continuous explanatory variables (physicochemical parameters) were included in a GLMM as a fixed effect, while “honey samples” from which replications were carried out were considered as a random effect (Pinheiro et al., 2015). The interaction of the two factors of “Bacterial strains × Floral origin” was also encompassed into the model using the function “lme” and the maximum likelihood (ML) method. The effect of each factor as well as their interaction was achieved using the function “anova” with the selection of likelihood ratio (LR) test with “marginal” type because our data were unbalanced with regards to the number of honey samples of each floral origin. The Akaike information criterion (AIC) was used to select the model with the best fit. Finally, the function “Effect” was applied for constructing “effect plots” of every single explanatory variable included in the GLMM.

## Results

### Physicochemical and pollen parameters of honey

The physicochemical analysis of the study honeys, generally, indicated a water content of 14.6%, with an acidic pH (4.38 ± 0.50), EC = 0.5 μs/cm, a HMF content = 82 mg/kg honey. The total sugars were 83% while the reducing sugars = 71%. On average, pollen density was 7.86 × 10^6^ grains/10 g of honey, while the concentration of proline = 525.5 ± 550.2 mg/kg honey (Table 1).

Table 1**Physicochemical parameters of honey samples harvested in the Algerian Sahara desert following their floral origins**.

**Table:** 

**Floral origin**	**Water content (%) [*F*_(7, 376)_ = 53.76, *P* < 0.001]**	**pH [*F*_(7, 376)_ = 4.83, *P* < 0.001]**	**Electrical conductivity (μS/cm) [*F*_(7, 376)_ = 11.60, *P* < 0.001]**	**HMF (mg/kg honey) [*F*_(7, 376)_ = 17.86, *P* < 0.001]**
*Astragalus gyzensis*	15.80 ± 0.50^d^ [15.2−16.4]	4.25 ± 0.00^a^ [4.25−4.3]	0.50 ± 0.00^ab^ [0.50−0.5]	123.67 ± 61.93^c^ [79.0−210.0]
*Diplotaxis harra*	13.60 ± 0.25^b^ [13.2−13.8]	4.46 ± 0.42^a^ [4.15−5.2]	0.46 ± 0.05^a^ [0.40−0.5]	79.00 ± 61.94^b^ [21.0−182.0]
*Eucalyptus globulus*	13.05 ± 1.02^ab^ [11.6−14.4]	4.31 ± 0.09^a^ [4.16−4.4]	0.46 ± 0.06^a^ [0.40−0.5]	28.50 ± 15.94^a^ [09.0−45.0]
Fabaceae sp.	12.20 ± 0.00^a^ [12.2−12.2]	4.18 ± 0.00^a^ [4.18−4.2]	0.46 ± 0.00^a^ [0.46−0.5]	184.0 ± 0.00^d^ [184.0−184.0]
*Prunus persica*	15.00 ± 0.00^cd^ [15.0−15.0]	5.05 ± 0.00^b^ [5.05−5.1]	0.57 ± 0.00^bc^ [0.57−0.6]	96.0 ± 0.00^bc^ [96.0−96.0]
*Retama retam*	15.67 ± 0.25^de^ [15.4−16.0]	4.23 ± 0.09^a^ [4.12−4.3]	0.49 ± 0.05^a^ [0.43−0.6]	71.67 ± 21.18^b^ [54.0−101.0]
*Ziziphus lotus*	15.12 ± 0.98^ce^ [14.2−17.0]	4.39 ± 0.21^a^ [4.19−4.7]	0.56 ± 0.08^c^ [0.46−0.6]	94.40 ± 69.96^bc^ [18.0−214.0]
Multifloral	14.87 ± 1.33^c^ [11.6−16.8]	4.39 ± 0.76^a^ [3.98−6.8]	0.49 ± 0.11^a^ [0.27−0.7]	77.45 ± 55.36^b^ [9.0−193.0]
All origins combined	14.61 ± 1.36 [11.6−17.0]	4.38 ± 0.50 [3.98−6.8]	0.50 ± 0.08 [0.27−0.7]	81.88 ± 59.93 [9.0−214.0]

**Table:** 

**Floral origin**	**Total sugars (%) [*F*_(7, 376)_ = 65.31, *P* < 0.001]**	**Reducing sugars (%) [*F*_(7, 376)_ = 24.06, *P* < 0.001]**	**Pollen density (grains × 10^6^/10 g) [*F*_(7, 376)_ = 5.73, *P* < 0.001]**	**Proline (mg/kg honey) [*F*_(7, 376)_ = 21.47, *P* < 0.001]**
*Astragalus gyzensis*	81.75 ± 0.62^a^ [81−82.5]	67.82 ± 1.48^ac^ [66.5−69.9]	6.22 ± 1.88^ab^ [4.64−8.8]	833.3 ± 430.0^b^ [237.9−1193]
*Diplotaxis harra*	84.06 ± 0.33^d^ [83.75−84.5]	75.43 ± 5.11^d^ [68.56−82.3]	2.19 ± 2.40^b^ [0.36−6.2]	135.9 ± 36.7^a^ [87.5−189]
*Eucalyptus globulus*	84.54 ± 0.79^d^ [83.25−85.4]	77.2 ± 7.67^d^ [65.54−86.8]	4.14 ± 3.73^b^ [0.18−9.7]	317.3 ± 444.4^a^ [27.0−1076]
Fabaceae sp.	86.23 ± 0.00^e^ [86.23−86.2]	63.39 ± 0.00^a^ [63.39−63.4]	12.92 ± 0.00^ab^ [12.92−12.9]	14.0 ± 0.0^a^ [14.0−14]
*Prunus persica*	82.50 ± 0.00^ac^ [82.5−82.5]	67.72 ± 0.00^ab^ [67.72−67.7]	1.06 ± 0.00^ab^ [1.06−1.1]	169.5 ± 0.0^a^ [169.5−170]
*Retama retam*	82.00 ± 0.21^ab^ [81.75−82.3]	66.03 ± 0.44^a^ [65.49−66.6]	6.34 ± 1.23^ab^ [4.78−7.7]	783.0 ± 225.7^b^ [468.5−950]
*Ziziphus lotus*	82.50 ± 0.94^bc^ [80.75−83.5]	71.67 ± 6.92^b^ [65.56−82.0]	2.26 ± 1.18^b^ [0.06−3.5]	261.0 ± 268.9^a^ [19.0−748]
Multifloral	82.80 ± 1.34^c^ [80.75−85.8]	69.82 ± 5.81^bc^ [63.43−84.1]	14.83 ± 28.60^a^ [0.64−103.6]	787.8 ± 682.2^b^ [19.0−2057]
All origins combined	83.05 ± 1.39 [80.75−86.2]	70.93 ± 6.54 [63.39−86.8]	7.86 ± 17.69 [0.06−103.6]	525.5 ± 550.2 [14.0−2057]

*Values of each parameter are given in means ± SD [range in square brackets], with the same superscript letters indicating no differences between means according to Tukey's post hoc tests, which followed One-way ANOVAs (F, F-value with df numerator and df denominator, P, P-value)*.

At the scale of pollinic composition, honeys dominated by Fabaceae sp. pollen contained less water (WC = 12.2%), while those dominated by *Astragalus gyzensis* were the most moisturized with WC = 15.8 ± 0.5%. The unifloral honey of *Prunus persica* had the highest EC value (0.57 μS/cm) and pH (5.05). The pH values of other types of honey ranged between 4.3 and 4.5. The unifloral honey of Fabaceae sp. showed the highest values of total sugars (86%) and HMF (184 mg/kg honey), but was the poorest in reducing sugars (63.4%). For the latter parameter, *Eucalyptus globulus* and *Diplotaxis harra* were the richest with 77 and 75%, respectively. The physicochemical parameters of multifloral honey were intermediate compared to other honeys except for pollen density where the maximum was recorded with 14.83 × 10^6^ grains/10 g of honey. The concentration of proline was higher in honeys of *A. gyzensis, Retama retam* and the multifloral, which represent the same homogenous group according to Tukey's test. Whereas the honey dominated by Fabaceae sp. pollen was the least rich in proline with only 14 mg/kg of honey. All ANOVAs revealed very significant differences (*P* < 0.001) between floral origins for all honey parameters, where homogeneous groups of Tukey's test differ between honeys from one parameter to another (Table 1).

### Antibacterial activity of honey according to floral origins

Overall, the antibacterial action of Saharan honeys differed from one bacterium to another. *E. coli* was the most sensitive species with a mean of inhibition diameter = 10.1 ± 4.7 mm (range: 0–24.9 mm) for all floral origins combined, while *C. perfringens* was the least sensitive with a mean activity of 3.9 ± 5.4 mm (range: 0–21.7 mm). The honeys tested against *B. subtilis* and *S. aureus* indicated an intermediate antibacterial activity between the two previous species with a mean = 8.0 ± 5.7 and 9.7 ± 1.5 mm, respectively.

Considering the floral origin of honeys, Fabaceae-pollen-based honey was the most effective with a mean of total activity = 13.5 ± 4.7 mm (min = 8.2, max = 21.7 mm). The activity of this type of honey was greater against *B*. *subtilis* (mean = 20 mm) and *E*. *coli* (mean = 15 mm). Other types of honeys showed moderate antimicrobial activities, with the following descending order: multifloral honey (9.1 ± 4.1 mm), *P. persica* (8.9 ± 7.6 mm), *Ziziphus lotus* (8.8 ± 5.1 mm), *D. harra* (7.2 ± 4.3 mm), *A. gyzensis* (6.9 ± 6.5 mm), *E. globulus* (5.5 ± 5.1 mm), and *R. retam* (5.3 ± 5.5 mm).

The use of trimethoprim-sulfamethoxazole as positive control against the strains of reference revealed inhibition activities of 24 mm for *E. coli* and 24.3 mm for *S. aureus*.

**Figure 2:**
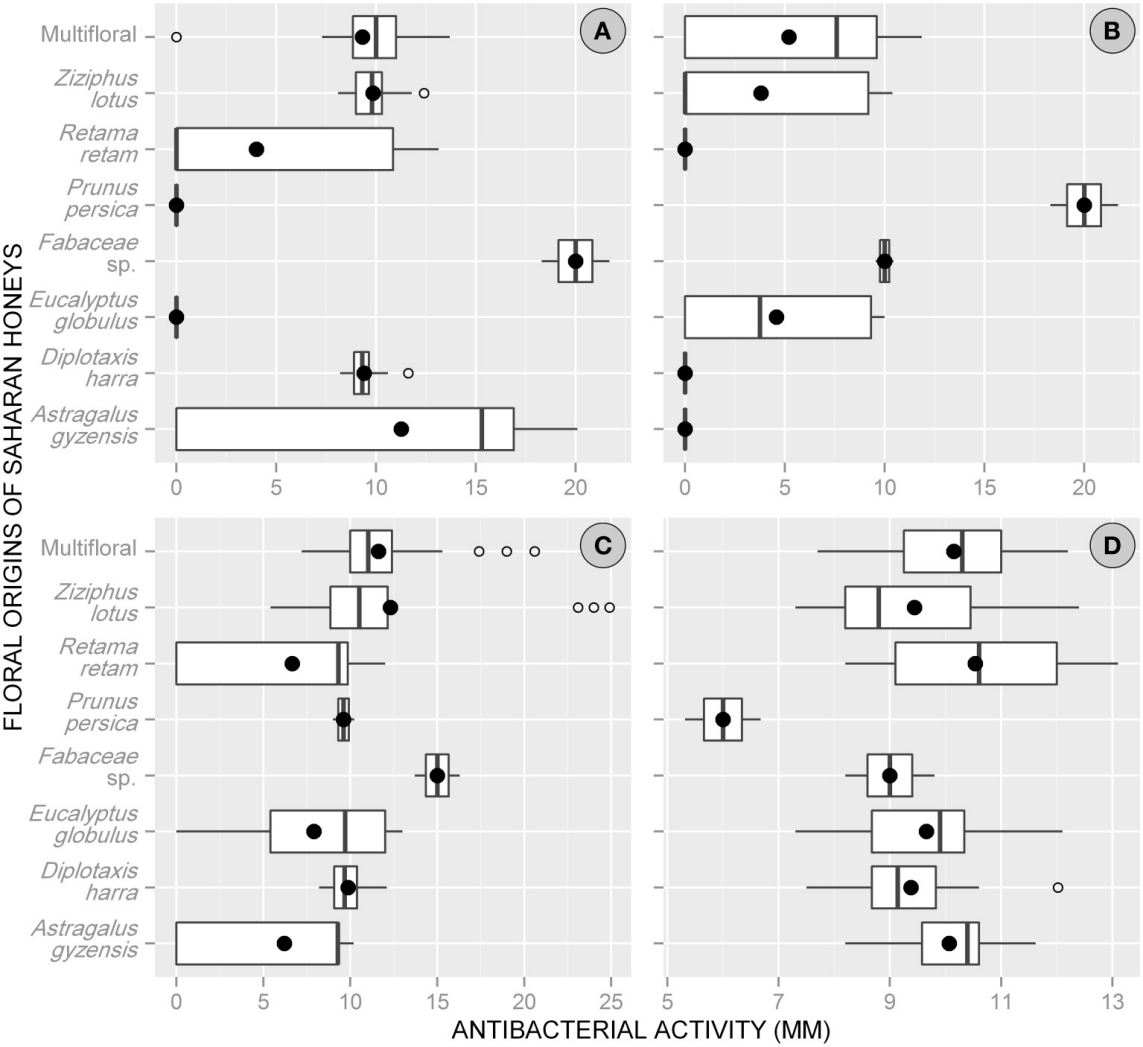
**Box plots displaying the variation of the average values (•) and quartiles of antibacterial activity (expressed via inhibition zone) among the floral origins of honeys collected from the Sahara Desert of Algeria**. (**A**, *Bacillus subtilis*; **B**, *Clostridium perfringens*; **C**, *Escherichia coli*; **D**, *Staphylococcus aureus*).

At the level of bacteria, all floral origins of honey showed an antimicrobial activity against *S*. *aureus* but with rather similar reactions (9–10.5 mm), except with *P. persica*-based honey, whose activity was only 6 mm. The bacteria *E*. *coli* experienced a greater inhibition effect when treated with honey of Fabaceae sp. (15.0 mm), *Z. lotus* (12.3 mm), and multifloral honey (11.6 mm). Whereas, a large variation in antibacterial activity of honeys was observed with both bacteria *B*. *subtilis* and *C*. *perfringens*. For *B*. *subtilis*, the antibacterial activity was higher with Fabaceae sp. honey (20 mm), but it was zero with those of *E. globulus* and *P. persica*. Similarly for *C*. *perfringens*, it was resistant toward honeys dominated by pollen of *A. gyzensis, D. harra*, and *R. retam*, while its growth was greatly reduced under treatment based on honey of *P. persica* (Figure 2).

The assessment of antimicrobial activity of different antibiotics were determined against the two reference strains by measuring diameters of the inhibition zones. *E. coli* and *S. aureus* were clearly sensitive to all tested antibiotics. The Sulfamides (Trimethoprim-sulfamethoxazole, Clindamycin, and Fusidic acid) and Aminozides antibiotics (Gentamicin) were the most active antibiotics against *E. coli*.

### Influence of honey parameters on antibacterial activity

Modeling the synergistic antimicrobial effects of honey parameters revealed that the inhibition zone “antimicrobial activity” was negatively associated with the bacteria *C. perfringens* and *E. coli* (*P* < 0.001 and *P* = 0.002, respectively). Whereas the variation of that activity was not significantly related in *B. subtilis* and *S. aureus*. The GLMM indicated that antimicrobial activity was higher (*P* = 0.003) in honeys dominated with Fabaceae sp. pollen compared to the other floral origins, where the diameter of inhibition zone significantly decreased when bacteria were treated respectively with multifloral honey (*P* = 0.049), *D. harra* (*P* = 0.030), *R. retam* (*P* < 0.001), *P. persica* (*P* < 0.001), *E. globulus* (*P* < 0.001). The inhibition zone was not significantly associated with honey of *Z. lotus* (*P* = 0.345) (Table 2).

**Table 2:** **Generalized linear mixed model (GLMM) testing the effects of physicochemical parameters of Saharan honeys from different botanical origins on pathogenic bacteria (Akaike information criterion = 2071.5)**.

**Variables**	**Estimate**	**2.5% CI**	**97.5% CI**	**SE**	***t*-value**	***P***	**Sig**
Intercept	−129.30	−264.45	5.86	69.0	−1.88	0.062	
*Clostridium perfringens*	−11.27	−14.42	−8.12	1.6	−7.01	< 0.001	^***^
*Escherichia coli*	−5.07	−8.22	−1.92	1.6	−3.15	0.002	^**^
*Staphylococcus aureus*	−1.20	−4.35	1.95	1.6	−0.75	0.456	ns
Fabaceae sp.	7.99	2.78	13.20	2.7	3.00	0.003	^**^
*Ziziphus lotus*	−1.41	−4.33	1.51	1.5	−0.95	0.345	ns
Multifloral honey	−2.59	−5.15	−0.02	1.3	−1.98	0.049	^*^
*Diplotaxis harra*	−3.73	−7.07	−0.38	1.7	−2.18	0.030	^*^
*Retama retam*	−7.65	−10.85	−4.45	1.6	−4.68	< 0.001	^***^
*Prunus persica*	−11.11	−15.65	−6.57	2.3	−4.80	< 0.001	^***^
*Eucalyptus globulus*	−13.24	−16.68	−9.80	1.8	−7.54	< 0.001	^***^
Water content (WC)	1.46	0.08	2.84	0.7	2.07	0.040	^*^
pH	0.79	−0.86	2.44	0.8	0.94	0.349	ns
Electrical conductivity (EC)	−13.60	−23.48	−3.72	5.0	−2.70	0.007	^**^
Hydroxymethylfurfural (HMF)	< 0.01	− 0.01	< 0.01	< 0.1	−1.03	0.303	ns
Total sugars	1.41	−0.06	2.88	0.7	1.88	0.060	ns
Reducing sugars	0.10	0.01	0.18	0.0	2.11	0.036	^*^
Pollen density	< 0.01	< 0.01	< 0.01	< 0.1	−1.52	0.128	ns
Proline	< 0.01	< 0.01	< 0.01	< 0.1	0.03	0.973	ns
*C*. *perfringens* × *D. harra*	1.87	−2.30	6.03	2.1	0.88	0.381	ns
*E*. *coli* × *D. harra*	5.54	1.37	9.71	2.1	2.61	0.010	^**^
*S*. *aureus* × *D. harra*	1.18	−2.99	5.34	2.1	0.55	0.580	ns
*C*. *perfringens* × *E. globulus*	15.84	11.67	20.01	2.1	7.45	< 0.001	^***^
*E*. *coli* × *E. globulus*	12.97	8.80	17.13	2.1	6.10	< 0.001	^***^
*S*. *aureus* × *E. globulus*	10.86	6.69	15.02	2.1	5.11	< 0.001	^***^
*C*. *perfringens* × Fabaceae sp.	1.27	−5.03	7.57	3.2	0.39	0.694	ns
*E*. *coli* × Fabaceae sp.	0.07	−6.23	6.37	3.2	0.02	0.983	ns
*S*. *aureus* × Fabaceae sp.	−9.80	−16.10	−3.50	3.2	−3.05	0.002	^**^
*C*. *perfringens* × *P. persica*	31.27	24.97	37.57	3.2	9.73	< 0.001	^***^
*E*. *coli* × *P. persica*	14.68	8.38	20.98	3.2	4.57	< 0.001	^***^
*S*. *aureus* × *P. persica*	7.20	0.90	13.50	3.2	2.24	0.026	^*^
*C*. *perfringens* × *R. retam*	7.27	2.81	11.72	2.3	3.20	0.002	^**^
*E*. *coli* × *R. retam*	7.73	3.27	12.18	2.3	3.40	< 0.001	^***^
*S*. *aureus* × *R. retam*	7.73	3.28	12.19	2.3	3.40	< 0.001	^***^
*C*. *perfringens* × *Z. lotus*	5.22	1.23	9.20	2.0	2.57	0.011	^*^
*E*. *coli* × *Z. lotus*	7.52	3.53	11.50	2.0	3.70	< 0.001	^***^
*S*. *aureus* × *Z. lotus*	0.79	−3.19	4.78	2.0	0.39	0.697	ns
*C*. *perfringens* × Multifloral	7.15	3.59	10.70	1.8	3.94	< 0.001	^***^
*E*. *coli* × Multifloral	7.37	3.82	10.92	1.8	4.06	< 0.001	^***^
*S*. *aureus* × Multifloral	2.03	−1.52	5.58	1.8	1.12	0.264	ns

*CI, confidence interval; SE, standard error; P, p-value; Sig, significance codes*;

*** *P < 0.001*,

** *P < 0.01*,

* *P < 0.05*,

*ns: not significant: P > 0.05*.

**Figure 3:**
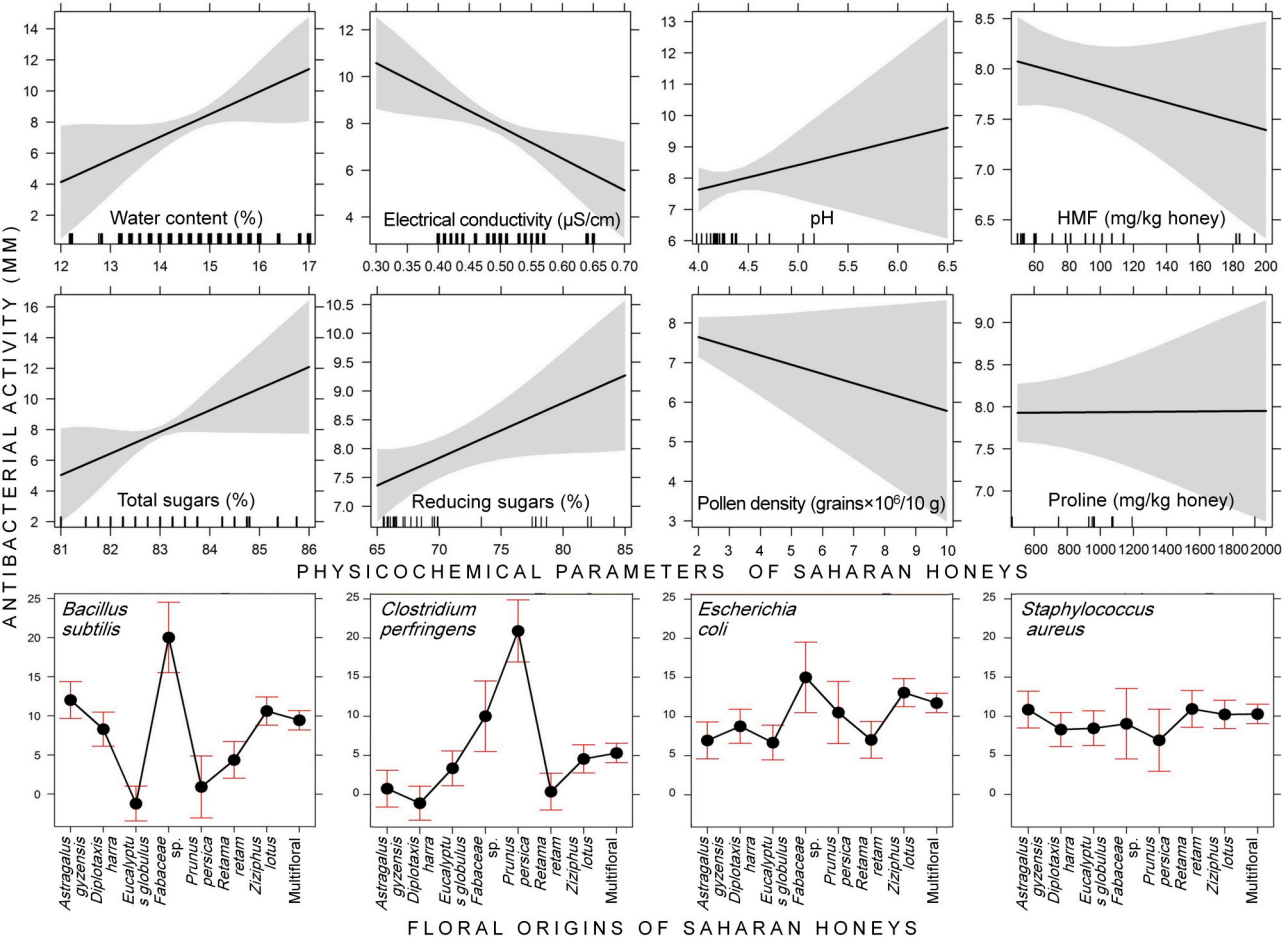
**Effect graphs constructed based on the generalized linear mixed model “GLMM” testing the effects of physicochemical parameters and floral origins of Saharan honeys on four bacteria species**. The antibacterial activity of honeys was expressed via the clear inhibition zone.

Regarding the physicochemical parameters of honey, the statistical model demonstrated that the antimicrobial activity increased with increasing water content (*P* = 0.040); whereas it significantly decreased with increasing electrical conductivity (EC) (*P* = 0.007). Moreover, the antimicrobial activity was positively correlated with reducing sugars (*P* = 0.036). The rest of parameters have no significant effect on the variation of inhibition zone diameter (Figure 3, Table 2).

The interaction of the two factors “Bacterial strains × Floral origin” showed that the antimicrobial effect of Saharan honeys against *C*. *perfringens* and *E*. *coli* was deemed positively related to honeys originated from *E. globulus, P. persica, R. retam, Z. lotus*, and multifloral origin. While the inhibition zone in *S*. *aureus* was associated negatively with Fabaceae sp. but positively with honeys of *E. globulus, R. retam*, and *P. persica* (Table 2).

The LR test of the GLMM revealed that there was a highly significant effect of the bacterial strains, floral origins and their interaction “Bacterial strains × Floral origins” (*P* < 0.001) on the variation of antimicrobial activity of Saharan honeys (Table 3). Moreover, water content, EC, and reducing sugars concentration in honey significantly affected the antimicrobial activity. However, the effects of pH, HMF, total sugars, pollen density, and proline content were not significant (*P* > 0.05).

**Table 3:** **Modeling the effects of physicochemical parameters of honeys with different floral origins and tested bacterial strains on the antimicrobial activity of honeys collected from the Sahara desert of Algeria**.

**Variables**	**Sum Sq**.	***Df***	***F***	***P***	**Sig**
Bacterial strains	2340.3	3	67.11	< 0.001	^***^
Floral origins	1207.8	7	14.84	< 0.001	^***^
Bacterial strains × Floral origins	2554.3	21	10.46	< 0.001	^***^
Water content (WC)	49.6	1	4.27	0.040	^*^
pH	10.2	1	0.88	0.349	ns
Electrical conductivity (EC)	84.6	1	7.28	0.007	^**^
Hydroxymethylfurfural (HMF)	12.4	1	1.06	0.303	ns
Total sugars	41.3	1	3.55	0.060	ns
Reducing sugars	51.5	1	4.43	0.036	^*^
Pollen density	27.0	1	2.32	0.128	ns
Proline	< 0.1	1	< 0.01	0.973	ns
Residuals	3998.9	344			

*Sum Sq., sum of squares; Df, degrees of freedom; F, F-statistic; P, P-value; Sig, significance codes*;

*** *P < 0.001*,

** *P < 0.01*,

* *P < 0.05*,

*ns, not significant: P > 0.05*.

## Discussion

This essay showed that both physicochemical properties and pollen composition of Saharan honey differ depending on their botanical origins. Thus, according to these two characteristics the antibacterial activity of these honeys varies between the tested bacteria. This may be explained by the fact that the antibacterial activity of honey essentially depends on the type of flowers from which bees gather nectar (Allen et al., 1991). But also the sensitivity/resistance of study strains influences that activity (Shahid et al., 2008), as it is the case of *E. coli* which reacted with high antibacterial activity values for both study honeys and antibiotics.

The significant variation in antimicrobial activity among the bacterial strains is assigned to the specificity of each bacterium, which reacts differently to honey parameters. According to Zaika (1988), Gram-positive bacteria are more resistant to essential oils than Gram-negative bacteria. This statement was however not confirmed by honey-related studies. Nevertheless, our honeys showed a higher antibacterial activity against *E. coli*, a Gram-negative bacterium, compared to the other three Gram-positive bacteria. Indeed, *S. aureus* resisted to several antibiotics so the resulted inhibition zones by honeys were slightly lower compared to those of *E. coli*. Though testing quantitatively a variety of each group species with a good number of isolates provides determine relevantly the activity trend of honeys. Despite that, our results are in agreement with the investigations of Shamala et al. (2002) in which honey showed a significant antibacterial activity against *E*. *coli* either *in vitro* and *in vivo* conditions. Additionally, the marked sensitivity of study bacteria to certain types of honey (e.g., with the origin of Fabaceae sp.) is probably linked to medicinal properties of the dominant flower from which honey was produced. These effective honeys can be used as an alternative to fight against some resistance strains.

Our results revealed that the overall antimicrobial activity increases with the content of water in honey. These findings are similar to those of honeys from New Zealand, where the antibacterial activity was found to be more effective at low concentrations of honey (Molan and Russell, 1988). This assumes that the antimicrobial activity of our honeys depends on the content of endogenous hydrogen peroxide, which is the main antibacterial agent in honey (Morse, 1986). In fact, the antibacterial potential of hydrogen peroxide results from the action of these highly reactive oxidizing molecules, which play the role of a “cleaning agent” attacking the cell membrane of microorganisms by producing free radicals that induce cell destruction. The latter cause damage to cell membrane lipids, isolating the cell, inhibiting the entry of nutrients, and the removal of waste material; thus triggering gradually slow death of the microorganism (Lu et al., 2005; Brudzynski, 2006; Erejuwa et al., 2012). Since water is essential to the oxidation process, hydrogen peroxide is typically produced in immature honeys in which water content is high. While in a ripe honey in which moisture content is low, glucose oxidase remains inactive so the oxidation process are limited. Thus, the honey contains a small amount of hydrogen peroxide insufficient to prevent bacterial growth unless water content increases (Bogdanov and Blumer, 2001). That perfectly explains the positive correlation between honey moisture content and the antimicrobial action obtained in the statistical model.

Furthermore, other molecules grouped under the name of non-peroxide inhibins can be the cause of the antibacterial action of honey; their origin is also the subject of lively discussions (Mavric et al., 2008; Mandal and Mandal, 2011). Some studies state that these molecules are of plant origin, while others declare that are added by bees when developing honey. The role of non-peroxide inhibins, often underestimated, is very important as they are at a large extent: insensitive to heat and light, and remains intact after honey storage for long periods (Bogdanov, 1984; Reybroeck et al., 2014).

The antimicrobial activity of Saharan honeys was more effective against bacteria when the honey has low EC. The latter is linked to the ionizable material content in which the mineral matter represents the essential. EC depends on the nature of the dissolved ions and their concentration (Rejsek, 2002), which in turn is linked to the botanical origin of honey and indirectly linked to various environmental conditions, including edaphic factors upon which melliferous plants substantially depend (Thasyvorlor and Manikis, 1995). This corroborates with our findings where the antimicrobial activity varied significantly between botanical origins, which are behind the significant change in EC.

Furthermore, according to Bonté and Desmoulière (2013), potassium salts represent almost half of honey inorganic materials, but there is also calcium, sodium, magnesium, copper, manganese, chlorine, sulfur, silicon, iron, and more than 30 trace elements. Minerals play an important role in biological systems, but can also cause harmful effects if their inputs exceed the recommended amounts (Tuzen and Soylak, 2005). Our findings imply that the factors that may affect the antibacterial activity of honey can have a redundant activity, or be mutually dependent, or even have antagonistic or synergistic activity against different bacterial species (Thasyvorlor and Manikis, 1995; Wahdan, 1998).

The positive correlation of the antimicrobial activity with reducing sugars is associated to the osmotic effect generated by high sugar concentration in honey. As honey is hypertonic, and due to the action of simple sugars on water contained in bacteria, it causes the lysis of the bacterial membrane, inhibition of the growth and then death of the microorganism (Couquet et al., 2013). For this parameter, our results are similar to those reported in Mandal and Mandal (2011).

Since the antibacterial activity was high with honeys originated from Fabaceae sp. *P. persica, Z. lotus* and multifloral honey, we speculate that these honeys contain a high content of hydrogen peroxide and even other non-peroxide inhibins such as lysozymes, flavonoids, aromatic acids, and volatile substances (Wahdan, 1998; Brudzynski, 2006; Montenegro and Mejías, 2013). Therefore, the use of sophisticated and complementary techniques enables detecting and quantifying accurately these compounds in different botanical origins of honey (Alzahrani et al., 2012). For example, for the analysis of phenolic acids and flavonoids, which depend on the floral origin of honey, the use of modern conventional techniques such as GC-MS and LC-MS allows to determine the floral origin of honey having the more effective use as an antimicrobial agent (Bertoncelj et al., 2007; Boukraâ, 2013). In addition, NMR allows the analysis of complex mixtures of natural products such as honey and thus the identification and quantification of various families of compounds regardless of their structure (Tiwari et al., 2015).

## Conclusion

This assay shows that Saharan honeys have multiple floral origins, which are causing differences in their physicochemical and pollinic characteristics. The GLMM revealed that antibacterial effect increases with increasing water content and reducing sugars in honey, while it decreases with increasing EC. These three parameters are the more relevant parameters that were correlated with antibacterial activity that differed significantly from one bacterium to another. *E. coli* was the most sensitive species while *C*. *perfringens* was the least sensitive. Honeys tested against *B. subtilis* and *S. aureus* indicated intermediate antibacterial activity.

In light of floral origins, our results suggest that Saharan honeys with the floral origin of Fabaceae sp. have a higher detrimental effect on bacteria compared to other spontaneous Saharan species, known for their common uses in traditional medicine such as *Zizyphus lotus* or *D. harra*. Most likely, this returns to the source of nectar collected from these species well-adapted to arid conditions. Yet, several factors, particularly the ecological ones, can affect the melliferous plants; thus additional research are required to fill the scientific gaps in this still virgin field of research in drylands.

## Author contributions

HL designed the study, collected honey samples, carried out all laboratory experiments (antibiotic tests, honey antibacterial assays, physicochemical, and pollinic analyses), and drafted the manuscript. LB contributed in the pollinic analysis. SB gave technical support and conceptual advice. TM helped in drafting and revision of the manuscript. SH and MM performed physicochemical analyses on honey samples. RH conducted the experiment of honey antibacterial tests. HC conceived the paper, analyzed and modeled statistically data, wrote and revised the article.

### Conflict of interest statement

The authors declare that the research was conducted in the absence of any commercial or financial relationships that could be construed as a potential conflict of interest.
